# The association between adverse childhood experiences and mental disorders among adolescents in Kenya, Indonesia, and Vietnam: Evidence from the National Adolescent Mental Health Surveys

**DOI:** 10.1186/s13034-025-00919-z

**Published:** 2025-07-31

**Authors:** Yohannes Dibaba Wado, Anne Njeri, Sally Atieno Odunga, Isaiah Akuku, Amirah Ellyza Wahdi, Shoshanna L. Fine, Astha Ramaiya, Mengmeng Li, Vu Manh Loi, Joemer C. Maravilla, James G. Scott, Holly E. Erskine, Caroline W. Kabiru

**Affiliations:** 1 African Population and Health Research Center, Nairobi, Kenya; 2 Center for Reproductive Health, Faculty of Medicine, Public Health, and Nursing, Universitas Gadjah Mada, Yogyakarta, Indonesia; 3 Department of Biostatistics, Epidemiology, and Population, Faculty of Medicine, Public Health, and Nursing, Universitas Gadjah Mada, Yogyakarta, Indonesia; 4 Department of Population, Family and Reproductive Health,, Johns Hopkins Bloomberg School of Public Health, Johns Hopkins University, Baltimore, MD USA; 5 Institute of Sociology, Vietnam Academy of Social Sciences, Hanoi, Vietnam; 6 School of Public Health, The University of Queensland, Herston, QLD Australia; 7 Queensland Centre for Mental Health Research, Wacol, QLD Australia; 8 Institute of Health Sciences and Nursing, Far Eastern University, Manila, Philippines; 9 Child Health Research Centre, The University of Queensland, South Brisbane, QLD Australia; 10 Child and Youth Mental Health Service, Children’s Health Queensland, South Brisbane, QLD Australia; 11 Institute for Health Metrics and Evaluation, University of Washington, Seattle, WA USA; 12 Department of Mental Health, , Johns Hopkins Bloomberg School of Public Health, Johns Hopkins University, Baltimore, MD USA

**Keywords:** Adverse childhood experiences, Adolescents, Mental disorders, Kenya, Indonesia, Vietnam

## Abstract

**Background:**

Few studies have examined the prevalence of adverse childhood experiences (ACEs) among adolescents living in low- and middle-income countries, and fewer assessed the association with mental disorders.

**Methods:**

We used data from nationally representative household surveys of mental disorders among adolescents aged 10–17 years conducted in Kenya, Indonesia, and Vietnam. The lifetime experience of 13 ACEs was measured using a self-administered questionnaire. Mental disorders were measured using a diagnostic instrument. The proportion of adolescents who endorsed each individual ACE, as well as those who endorsed one or more and four or more ACEs, was calculated. Multivariable logistic regression was used to examine the associations between the number of ACEs endorsed and any mental disorder in the past 12 months, after adjusting for demographic characteristics and primary caregiver mental health.

**Results:**

The prevalence of experiencing at least one ACE was evident among adolescents in all three countries, with Kenya (65.8%, 95% CI: 63.0–68.5) demonstrating significantly higher prevalence than Indonesia (40.2%, 95% CI: 36.4–44.1) and Vietnam (36.9%, 95% CI: 33.1–40.8). Significant differences were seen between all countries in the prevalence of adolescents who experienced four or more ACEs (Kenya: 19.3%, 95% CI: 17.5–21.2; Indonesia: 7.6%, 95% CI: 6.3–9.1; Vietnam: 5.2%, 95% CI: 4.2–6.3). The odds of experiencing a mental disorder in the past 12 months increased as the number of ACEs increased in all three countries. This was most apparent among those experiencing four or more ACEs, who had the highest odds of any mental disorder in the past 12 months as compared to those reporting no ACEs (Kenya: aOR 4.57, 95% CI: 3.35–6.23; Indonesia: aOR 11.10, 95% CI: 6.24–19. 73; Vietnam: aOR 10.30, 95% CI: 5.96–17.82).

**Conclusion:**

The current study demonstrated that ACEs are common among adolescents in Kenya, Indonesia, and Vietnam, and are significantly associated with mental disorders in all three countries. The prevention of ACEs may be a key avenue for reducing the risk of mental disorders in adolescence.

**Supplementary Information:**

The online version contains supplementary material available at 10.1186/s13034-025-00919-z.

## Background

Mental disorders are a major contributor to the burden of disease among adolescents globally [1], accounting for approximately 13% of the disease burden in young people aged 10–19 years [2]. The prevalence, persistence, and severity of mental disorders, both during adolescence and later in life, are influenced by social, economic, environmental, and genetic factors [3, 4]. Research has highlighted how early exposure to adversity is associated with higher prevalence of mental disorders in adolescence, which may further persist into adulthood [3, 5, 6]. When occurring early in life, exposures to adverse events are referred to as adverse childhood experiences (ACEs). ACEs include sexual, physical, or emotional abuse, childhood neglect, household dysfunction (such as living in a household with a person with a drinking problem), deprivations and poverty, and other traumatic events (such as death of a parent) [3, 7].

The available literature reporting the prevalence of ACEs, as well as their association with mental health, comes largely from high-income countries (HICs). Studies from HICs have found that ACEs are common, with prevalence estimates ranging from 40 to 65% for those experiencing one or more ACEs and 8–20% for those experiencing four or more ACEs [5–7]. However, many studies are based on retrospective reports of ACEs among adults, with far fewer studies researching ACEs reported by adolescents [8]. A systematic review of studies from both HICs and low- and middle-income countries (LMICs) assessing ACEs among school-aged youth (younger than 18 years) found that the prevalence of one or more ACEs varied widely from 41 to 97% [9]. Prevalence studies of ACEs in adolescent populations in HICs vary greatly in terms of ACEs measured and are often limited to school-based samples. As such, methodological differences between studies generally impedes direct comparisons [9, 10]. Like prevalence studies of ACEs, many of the studies of associated health outcomes are based on adults’ retrospective reports of ACEs, and little is known about the impact of ACEs during adolescence.

Comparatively few studies have examined the prevalence of adverse childhood experiences (ACEs) among adolescents living in LMICs. A study from Indonesia among university students aged 18–20 years reported that more than 45% had experienced at least one ACE before they were 18 years [11]. In Kenya, a cross-sectional study among adolescents aged 12–19 years in the informal settlements of Nairobi found that 54% had experienced at least one ACE, while 18% reported three or more ACEs [12]. In Vietnam, a study conducted among high school students in two provinces found that approximately 74% of students reported experiencing at least one ACE, and more than 25% reported experiencing three or more ACEs [13]. The few available studies from LMICs reporting the prevalence of ACEs have similar limitations as studies from HICs. Most of the available studies are not representative of the broader population (e.g., are either based on school or health facility samples), or cover smaller geographic units [11, 12, 14].

In addition to evidence reporting the prevalence of ACEs, research shows exposure to multiple ACEs increases the risk of mental disorders [6, 15]– [17]. A systematic review and meta-analysis of the effects of multiple ACEs on health showed that individuals who had experienced four or more ACEs had a three- to six-fold increase in the risk of mental illness and problematic alcohol use, as compared with individuals reporting no ACEs [6]. A prospective study in the United States found that ACEs were significantly associated with incident depressive symptoms, substance use, and antisocial behavior after two year follow-up [18]. These findings are supported by evidence from systematic reviews of studies from both LMICS and HICs [19–21].

The few studies from LMICs reporting the association between ACEs and mental health outcomes have found similar results to that of evidence from HICs. For example, a study by Blum and colleagues examined the association between cumulative ACEs and depressive symptoms in 14 urban communities in LMICs using data from the Global Early Adolescent Study [7]. This study found that adolescents (age 10–14) who experienced one or more ACEs were at 88% more likely to have more than three depressive symptoms [7]. A study of adolescents from Nairobi slums found that childhood adversity was positively and significantly associated with delinquency [12]. However, many studies from LMICs are small scale, not nationally representative, and are narrow in focus, for example limiting their examination to the association between ACEs and a single mental disorder [7, 11, 12]. In addition, previous studies of ACEs in LMICs and HICs have largely used symptom scales and not diagnostic instruments to assess mental health outcomes.

More research is required to shed light on the prevalence of ACEs and their associations with mental disorders in LMICs, where the majority of the world’s adolescents live. Given that the prevalence and profile of ACEs may differ from HICs [3, 6], this information is necessary for policymakers, programmers, and researchers in LMICs to develop effective prevention programs to reduce exposure to ACEs and promote adolescent mental health and wellbeing.

In this study, we utilize data from the National Adolescent Mental Health Surveys (NAMHS) [22] to examine the prevalence of ACEs experienced by adolescents aged 10–17 years in Kenya, Indonesia, and Vietnam. Further, data from these nationally representative surveys are used to analyze the association between ACEs and mental disorders among adolescents in these three countries.

## Methods

### Sample

NAMHS employed a stratified multistage cluster sample design to generate a nationally representative sample of adolescents aged 10–17 years and their primary caregiver in Kenya, Indonesia, and Vietnam, respectively. NAMHS was conducted in these three countries due to their lack of data on adolescent mental health, as well as interest in adolescent mental health within these countries by in country and external stakeholders. More detailed rationale for the choice of the three countries, the study design, sample size, and sampling procedures of NAMHS have been published elsewhere [22, 23].

Data were collected in 2021 by trained lay interviewers who interviewed the adolescent and their primary caregiver. In each of the three countries, households were randomly selected after conducting household listing in the sampled enumeration areas (EAs), and one adolescent was randomly selected per household. The survey excluded adolescents if they did not live in the sampled household most of the time (less than 50% of the time), if they were married or living alone, if they did not speak the languages used for the interviews (Kiswahili or English in Kenya, Bahasa in Indonesia and Vietnamese in Vietnam) and if they were unable to participate in the study due to severe physical or cognitive impairment. Consent was obtained from the primary caregiver for both themselves and their adolescent to participate; and assent from the adolescent to participate in the study. All caregiver and adolescent interviews were administered separately in a private place. Interviewers were trained to follow and apply a structured distress protocol to manage any instances where a participant became distressed during the interview. This distress protocol was developed specifically for NAMHS and then adapted to each country’s setting. There were no reported instances where the distress protocol was enacted due to distress related to the ACEs questions which is in line with the experience of surveys including similar questions where any distress experienced was generally minimal and transient [24, 25]. Additionally, all participants were given a mental health services information sheet which detailed different services and support mechanisms that the participant could access after the interview. These information sheets were developed by each country and, to the fullest extent feasible, tailored for a given location i.e., to include local services as well as countrywide services. Further information about all study processes, including the distress protocol and mental health services information sheets, has been published previously [23].

Final sample sizes (and response rates) were 5,155 (98%) in Kenya, 5,664 (92%) in Indonesia, and 5,996 (81%) in Vietnam, respectively.

### Measures

#### Adverse childhood experiences

The measure of ACEs utilized in NAMHS was adapted from the WHO ACEs International Questionnaire [26]. It has previously been used in adolescent populations in LMICs by the Global Early Adolescent Study (GEAS) [7]. The ACEs measure contained 13 questions, with seven questions focused on child maltreatment (physical abuse, emotional abuse, physical neglect, emotional neglect [2 items], and sexual abuse [2 items] and six questions related to household level challenges (i.e., parental substance abuse, parental emotional distress, domestic violence, parental incarceration, and household instability [2 items]. The recall period for these questions was lifetime (i.e., ‘ever’). In NAMHS, the ACEs questions were answered by the adolescent who self-administered these questions (i.e., answering questions on the tablet/smartphone) rather than being asked by an interviewer. This approach was informed by previous literature, which found differences in responses to sensitive questions between interviewer- and self-administered methods [27, 28], and was therefore utilized to ensure greater accuracy and lessen any potential burden of disclosing, noting that the interviewer was there for support where needed. For each ACEs question, adolescents answered ‘Yes’, ‘No’, ‘Don’t know’, or ‘Prefer not to say’.

#### Mental disorders

Mental disorders were defined according to the Diagnostic and Statistical Manual of Mental Disorders, Fifth Edition (DSM-5) [29] measured by the Diagnostic Interview Schedule for Children, Version 5 (DISC-5) [30]. The DISC-5 is a standardized diagnostic instrument designed to be administered by trained ‘lay’ interviewers—individuals who do not have any clinical training but who are trained on the DISC-5. NAMHS measured the prevalence of six mental disorders in adolescents: social phobia, generalized anxiety disorder, major depressive disorder, conduct disorder, posttraumatic stress disorder, and attention-deficit/hyperactivity disorder (ADHD).

In NAMHS, all DISC-5 modules (i.e., measures of individual disorders) were administered to the adolescent except for ADHD (which was asked of the primary caregiver). The DISC-5 assessed for the presence of the selected mental disorders during the past 12 months. For the purpose of this study, mental disorders were grouped into a single category of any mental disorder in the past 12 months.

#### Demographics

Demographic information pertaining to both the adolescent and the primary caregiver was collected. All demographic information was reported by the primary caregiver except for urbanicity, which was determined based on household location. This included the age and sex of the adolescent, household wealth, and parental mental health. Household wealth was measured by the wealth index, which was analyzed according to standardized methodology that has been comprehensively detailed in a previous publication [23]. In brief, the household wealth index is constructed from the household’s ownership of selected assets, such as televisions and bicycles, materials used for housing construction, and types of water access and sanitation facilities. It is derived using principal component analysis following the methodology used in Demographic and Health Surveys (DHS) [31]. In addition to demographic information, the mental health of the primary caregiver was also included in the analyses. This was measured by the Patient Health Questionnaire-9 (PHQ-9) and Generalized Anxiety Disorder-7 (GAD-7), which were administered to the primary caregiver to assess their depressive symptoms and anxiety symptoms, respectively [23]. The PHQ-9 and GAD-7 scores were generated and categorized based on established thresholds [32, 33].

### Statistical approach

Data were analyzed using Stata version 17 [34]. The proportion of participants endorsing each of the 13 ACEs questions was calculated, and the 13 ACEs items were used throughout all analyses. Participants who gave a non-meaningful response (i.e., ‘Don’t know’ or ‘Prefer not to say’) to the respective question were omitted (see Table S1 in Additional File 1). The prevalence of participants endorsing any ACE and those endorsing four or more ACEs was calculated out of the 13 ACE questions (the prevalence of participants endorsing no ACEs, one ACE, two ACEs, three ACEs, and four or more ACEs is available in Table S2 in Additional File 1). In addition, the 13 ACE questions were also grouped into ten domains, following the domains defined in previous studies [35, 36]. For domains assessed by two questions (e.g., sexual abuse), endorsement of one or both questions counted as endorsement of the domain. Participants who gave a non-meaningful response to both questions within a given domain were omitted from the analysis (see Table S1 in Additional File 1).

For each country, results are presented as weighted proportions with associated 95% confidence intervals (CIs). Sampling weights applied in each country were generated using inverse probability weighting, which considered the complex sampling design in each country [23]. Stata’s survey (svy) function was used to account for the complex sampling design and adjust the standard errors for the multi-stage design, allowing Stata to correctly calculate the within and between cluster correlations and the survey design effects.

Bivariate analyses were conducted to examine the prevalence of four or more ACEs (i.e., of the 13 individual ACE questions) by selected demographic characteristics (age, sex, urbanicity, and wealth quintile). The prevalence of any mental disorder in the past 12 months was calculated within each of the five mutually exclusive categories of ACEs endorsement (i.e., none, one, two, three, four or more). Multivariable logistic regression analysis was used to examine associations between the five categories of ACEs and any mental disorder in the past 12 months after adjusting for demographic characteristics– age (10–14 years, 15–17 years), sex (male, female), urbanicity (urban, rural), and household wealth (as wealth index quintiles) and caregiver’s mental health status.

## Results

### Prevalence of adverse childhood experiences 

As shown in Table 1, the prevalence of the different ACEs, both individual and by domain, differed significantly between the countries. In Kenya, household instability (39.7%, 95% CI: 36.9–42.4), emotional abuse (24.6%, 95% CI: 22.8–26.6), and emotional neglect (21.1%, 95% CI: 19.3–22.9) were the most prevalent ACEs domains reported by adolescents. In Indonesia, emotional neglect (18.4%, 95% CI: 16.1–21.0), and emotional abuse (15.9%, 95% CI: 13.7–18.5) were the most prevalent ACE domains. Similarly, in Vietnam, emotional abuse (14.9%, 95% CI: 12.5–17.5) and emotional neglect (14.2%, 95% CI: 12.4–16.3) were the most commonly endorsed ACEs domains by adolescents (Table 1).

Kenya had significantly higher prevalence of adolescents who had experienced one or more ACEs (65.8%, 95% CI: 63.0-68.5) than Indonesia (40.2%, 95% CI: 36.4–44.1) and Vietnam (36.9%, 95% CI: 33.1–40.8). Among those reporting four or more ACEs, statistically significant differences were seen between Kenya and the other two countries, with Kenya having the highest prevalence (19.3%, 95% CI: 17.5–21.2) followed by Indonesia (7.6%, 95% CI: 6.3–9.1) and Vietnam (5.2%, 95% CI: 4.2–6.3) (Table 1).

**Table 1:** Prevalence of adverse childhood experiences (ACEs) among adolescents in Kenya, Indonesia, and Vietnam

ACEs domain Individual ACE item/s	Kenya % (95% CI)	Indonesia % (95% CI)	Vietnam % (95% CI)
**Physical abuse** 1. Ever scared that your parents/other adults were going to hurt you badly	11.9 (10.6–13.4)	12.3 (10.6–14.1)	7.3 (5.1–10.3)
**Emotional abuse** 2. Ever scared/felt really bad because grown-ups called you names	24.6 (22.8–26.6)	15.9 (13.7–18.5)	14.9 (12.5–17.5)
**Neglect** 3. Ever been a time in your life when you were totally on your own	18.7 (16.9–20.7)	9.2 (7.7–11.0)	8.4 (6.9–10.2)
Emotional neglect*	**21.1 (19.3–22.9)**	**18.4 (16.1–21.0)**	**14.2 (12.4–16.3)**
4. Ever felt like you are not loved or cared about	16.4 (15.0–18.0)	15.6 (13.5–18.0)	10.9 (9.4–12.6)
5. Ever felt like you have no one that protects you	12.0 (10.8–13.5)	8.8 (7.5–10.4)	8.7 (7.5–10.2)
Sexual abuse*	**11.0 (9.8–12.3)**	**5.8 (4.8-7.0)**	**1.9 (1.5–2.5)**
6. Ever touched by an adult in your private parts except when bathing	7.9 (6.9–9.1)	4.6 (3.7–5.7)	1.6 (1.2–2.2)
7. Ever had an adult attempt to or forced you to have sexual intercourse	5.4 (4.7–6.2)	2.2 (1.6–2.8)	0.6 (0.4–0.9)
**Parental substance use** 8. Ever had parents who drank too much alcohol/used drugs and were abusive	6.4 (5.5–7.4)	1.4 (1.0-2.1)	2.4 (1.0-5.5)
**Poor parental mental health** 9. Ever saw mother/father so sad that they couldn’t take care of you	17.5 (15.8–19.4)	7.7 (6.5–9.2)	8.6 (7.0-10.6)
**Domestic violence** 10. Ever saw your mother being hit, beaten, or threatened	14.8 (13.4–16.4)	3.7 (3.0-4.7)	5.5 (4.5–6.7)
**Parental incarceration** 11. Ever had either of parents be in prison/jail	7.9 (6.8–9.2)	0.6 (0.4-1.0)	1.1 (0.7–1.6)
Household instability*	**39.7 (36.9–42.4)**	**12.0 (10.1–14.2)**	**8.3 (6.8–10.0)**
12. Ever had family forced to leave home	5.6 (4.9–6.4)	1.8 (1.3–2.4)	1.0 (0.7–1.5)
13. Ever a time when family did not have enough food because of money	38.1 (35.3–41.0)	11.6 (9.6–13.9)	8.0 (6.5–9.9)
**One or more ACEs (out of 13 individual ACEs)**	65.8 (63.0-68.5)	40.2 (36.4–44.1)	36.9 (33.1–40.8)
**Four or more ACE (out of 13 individual ACEs)**	19.3 (17.5–21.2)	7.6 (6.3–9.1)	5.2 (4.2–6.3)

Note: Unweighted numerators and denominators are presented in Additional File 1.

*Bold percentages indicate prevalence of domains that were assessed by two questions.

95% CI = 95% confidence interval

As shown in Table 2, the prevalence of adolescents reporting four or more ACEs in Kenya was significantly higher among older (15–17 years) adolescents compared to younger (10–14 years) adolescents. However, there was no statistically significant difference in ACEs prevalence by age for Indonesia and Vietnam. Similarly, no significant differences in ACEs were observed by sex and urbanicity, although ACEs prevalence varied by household wealth in Indonesia and Kenya (Table 2). In Indonesia, the prevalence of four or more ACEs decreased as wealth increased. In Kenya, an inverse-U shaped association was seen whereby the prevalence of four or more ACEs was significantly higher in the third wealth quintile compared to the lowest and highest wealth quintiles (Table 2).

**Table 2:** Prevalence of four or more adverse childhood experiences (ACEs) by demographic characteristics among adolescents in Kenya, Indonesia, and Vietnam

Demographic characteristic	Kenya, % (95% CI)	Indonesia, % (95% CI)	Vietnam, % (95% CI)
Age group			
10–14 years	16.7 (14.9–18.7)	7.1 (5.7–8.7)	4.5 (3.6–5.8)
15–17 years	24.4 (21.3–27.8)	8.6 (7.0-10.6)	6.4 (4.8–8.4)
Sex			
Male	19.3 (17.0-21.7)	6.9 (5.5–8.6)	5.2 (3.9–6.9)
Female	19.2 (17.0-21.6)	8.3 (6.7–10.3)	5.1 (3.9–6.7)
Residence			
Urban	20.5 (18.0-23.3)	7.6 (6.1–9.5)	6.0 (4.3–8.3)
Rural	18.5 (16.1–21.2)	7.5 (5.4–10.3)	4.8 (3.7–6.2)
Wealth quintile			
1 (Least)	16.7 (12.6–21.8)	12.7 (10.0-16.1)	5.8 (4.1–8.1)
2	21.0 (17.8–24.6)	6.9 (5.0-9.4)	4.2 (2.6–6.6)
3	22.9 (19.3–26.9)	7.8 (5.8–10.3)	6.4 (4.6–8.7)
4	21.7 (18.9–24.8)	6.4 (4.6–8.8)	5.8 (3.8–8.8)
5 (Most)	13.9 (11.4–16.9)	3.3 (2.1–5.2)	3.4 (2.3–5.1)

Note: Four or more ACEs out of 13 individual ACE items.

95% CI = 95% confidence interval

### Association between adverse childhood experiences and mental disorders

Table 3 shows the prevalence of any mental disorder in the past 12 months among those experiencing none, one, two, three, and four or more ACEs. Across all three countries, the prevalence of any mental disorder in the past 12 months increased as the number of ACEs increased. In Kenya, there was a four-fold difference in the prevalence of any mental disorder between those who had not experienced any ACEs (6.0%, 95% CI: 4.8–7.5) and those who had experienced four or more ACEs (25.8%, 95% CI: 22.4–29.5). In Indonesia, a nine-fold difference was seen between the same groups (none: 2.4%, 95% CI: 1.5–3.8; four or more ACEs: 22.3%, 95% CI: 17.0-28.7) with the same magnitude differences also seen in Vietnam (none: 1.8%, 95% CI: 1.3–2.5; four or more ACEs: 17.0%, 95% CI: 11.7–24.1).

**Table 3:** Prevalence of any mental disorder in the past 12 months by number of adverse childhood experiences among adolescents in Kenya, Indonesia, and Vietnam

Number of ACEs	Any mental disorder in the past 12 months		
	Kenya, % (95% CI)	Indonesia, % (95% CI)	Vietnam, % (95% CI)
None	6.0 (4.8–7.5)	2.4 (1.5–3.8)	1.8 (1.3–2.5)
1	9.5 (7.6–11.9)	5.3 (3.8–7.4)	1.9 (1.1–3.2)
2	11.4 (8.8–14.5)	10.2 (6.9–14.8)	7.1 (5.1–9.9)
3	13.9 (11.1–17.4)	7.9 (4.4–13.7)	7.5 (4.6–12.1)
4 or more	25.8 (22.4–29.5)	22.3 (17.0-28.7)	17.0 (11.7–24.1)

Note: Out of a possible 13 individual ACEs.

95% CI = 95% confidence interval

Table 4 shows the adjusted odds ratios (aORs) for the association between the number of ACEs and any mental disorder in the past 12 months among adolescents. The odds of having a mental disorder increased as the number of ACEs increased in all three countries (Table 4). In Kenya, compared to those reporting no ACEs, the aORs increased from 1.52 (95% CI: 1.10–2.10) among those who experienced one ACE to 4.57 (95% CI: 3.35–6.23) among those who reported four or more ACEs. Similarly, the odds of experiencing any mental disorder in the past 12 months increased as the number of ACEs reported increased in Indonesia (one ACE: 2.18, 95% CI: 1.20–3.96; four or more ACEs: 11.10, 95% CI: 6.24–19.73) and Vietnam (one ACE: 0.94, 95% CI: 0.49–1.81; four or more ACEs: 10.30, 95% CI: 5.96–17.82) (Table 4).

**Table 4:** Unadjusted and adjusted odds ratios for the association between number of adverse childhood events (ACEs) and any mental disorder in the past 12 months among adolescents in Kenya, Indonesia, and Vietnam

Number of ACEs	Kenya		Indonesia		Vietnam	
	OR (95% CI)	aOR (95% CI)	OR (95% CI)	aOR (95% CI)	OR (95% CI)	aOR (95% CI)
None	Ref.	Ref.	Ref.	Ref.	Ref.	Ref.
1	**1.64** **(1.18–2.27)**	**1.52** **(1.10–2.10)**	**2.32** **(1.34–4.01)**	**2.18** **(1.20–3.96)**	1.07 (0.57–2.01)	0.94 (0.49–1.81)
2	**2.00** **(1.43–2.80)**	**1.68** **(1.18–2.41)**	**4.68** **(2.52–8.69)**	**4.61** **(2.46–8.64)**	**4.23** **(2.63–6.80)**	**3.38** **(2.00–5.70)**
3	**2.52** **(1.74–3.65)**	**2.18** **(1.50–3.17)**	**3.54** **(1.64–7.67)**	**3.19** **(1.51–6.70)**	**4.49** **(2.46–8.20)**	**4.01** **(2.06–7.79)**
4 or more	**5.41** **(4.03–7.28)**	**4.57** **(3.35–6.23)**	**11.85** **(7.08–19.82)**	**11.10** **(6.24–19.73)**	**11.29** **(6.71–19.01)**	**10.30** **(5.96–17.82)**

Note: Out of 13 individual ACE items. Adjusted for adolescent age, adolescent sex, urbanicity, household wealth, and primary caregiver mental health.

OR = Unadjusted odds ratio; aOR = adjusted odds ratio; 95% CI = 95% confidence interval.

Bold estimates indicate statistical significance at *p* < 0.05.

## Discussion

This study used nationally representative surveys of adolescents aged 10–17 years from Kenya, Indonesia, and Vietnam to report the prevalence of ACEs and their association with mental disorders. ACEs were measured using the WHO ACEs International Questionnaire although it did not include experiences beyond the family and social context such as witnessing community violence, exposure to war or collective violence and bullying. Accordingly, NAMHS showed that substantial proportions of adolescents in each country endorsed at least one ACE; and this differed by country, from 65.8% in Kenya, to 40.2% in Indonesia and 36.9% in Vietnam. In Kenya, nearly one in five adolescents (19.3%) reported experiencing four or more ACEs compared to less than one in ten adolescents in Indonesia (7.6%) and Vietnam (5.2%). Interestingly, NAMHS also showed that the overall 12-month prevalence of any mental disorder among adolescents was 12.1%, 5.5% and 3.3% in Kenya, Indonesia and Vietnam respectively [23], meaning that the patterns of prevalence seen between countries for ACEs broadly followed that of mental disorders.

Although comparison with previous studies is difficult due to different methods for measuring ACEs, these rates are consistent with studies from HICs [9, 12, 18]. The prevalence of one or more ACEs in Kenya (65.8%) falls in the range reported in a systematic review of studies (ranging from 41 to 97%) among school-aged youth that also included studies from LMICs [9]. The equivalent proportion of adolescents in Indonesia and Vietnam is slightly less than previously reported [9]. Although most previous studies from LMICs report higher prevalence, these were focused on specific population groups or small geographic areas and are generally not comparable with the current study because they are not representative of the general population. The few studies from Kenya sampled the informal settlements and reported higher prevalence of ACEs among adolescents and adults [12, 37, 38]. Moreover, most of the studies from Indonesia and Vietnam were university- or school-based and reported higher prevalence of ACEs than the current study [11, 13, 39]. Nonetheless, the current study shows that a significant proportion of adolescents in LMICs have exposure to ACEs, albeit with substantial variation between the three countries. The difference in the prevalence of ACEs between Kenya and the other two countries is inexplicable without a close examination of structural factors such as poverty, security, and the social environment. While we ensured NAMHS methodology was applied consistently across the three countries, it is possible that the differences relate to differences in the contextual environmental and social factors that predispose children to ACEs.

The type of ACEs experienced by adolescents varied cross-nationally. Emotional abuse and neglect were among the most common ACEs reported in all three countries. This finding is in line with other national surveys such as the Violence Against Children Surveys (VACS). In Kenya, for instance, the 2019 VACS showed that about 31% of females and males reported childhood emotional violence, that is experienced emotional violence before age 18 [40]. Previous studies have shown that emotional abuse is particularly harmful to mental health across the lifespan [17, 41]. For instance, a national study in Australia found that emotional abuse was consistently and independently associated with increased odds of mental disorders [17]. The high rates of emotional abuse observed cross-nationally in the current study provide an opportunity for public health interventions that might reduce mental disorder prevalence. Additionally, household instability, such as food insecurity, was reported by a high proportion of adolescents in Kenya (39.7%). The reported level of household challenges reflects the high level of poverty and food insecurity in Kenya. For instance, the 2021 Kenya Continuous Household Survey (KCHS) showed an overall national poverty headcount rate of 38.6% and a food poverty headcount of 30.5% [42]. The findings indicate that poverty-related childhood adversity should be considered in designing interventions to improve mental health.

In general, exposure to ACEs did not vary significantly by sex, urbanicity, or household wealth, which suggests that the vulnerability of adolescents to ACEs cuts across demographic and socioeconomic lines. While several previous studies have reported differences by sex [7, 43, 44], with females being more vulnerable to certain ACEs such as sexual abuse, no difference in the prevalence of four or more ACEs was seen between the sexes. The relationship between household wealth and ACEs varied across the countries. Some studies have found that both low socio-economic status and ACEs independently increase the risk of mental disorders in children [45, 46], while other studies have suggested that ACEs may mediate the relationship between socio-economic status and later life health outcomes [47]. The disparity in findings related to ACEs and household wealth in the current study further demonstrate the likely complexity of this relationship.

Consistent with previous studies from HICs and LMICs [6, 7, 48], the present study found that exposure to multiple ACEs is significantly associated with mental disorders among adolescents. Across all three countries, the prevalence of mental disorders increased as the number of ACEs experienced increased, indicating a dose-response relationship. The mechanisms by which ACEs contribute to mental disorders is less clear but may include activation of the body’s stress response with sensitization of neurobiological systems, making an individual more vulnerable to mental illness [49]. Additionally, ACEs can lead to psychological changes in children such as distrust of others and biased emotional processing, which impact on learning and formation of stable friendships and other relationships [50]. These and other pathways are purported to underpin a causal relationship between ACEs and mental disorders. Our study focused on the association between ACEs and mental health but the literature on ACEs shows that ACEs is strongly associated with various aspects of health and development [6, 15]. A recent review by Bhutta and colleagues assessed the mechanisms linking adverse childhood experiences to health and development and highlighted how social environments associated with ACES, beginning from the prenatal stage, affect the health and development of children and adolescents, and lifelong health [51].

There are certain limitations that must be considered when interpreting the findings of the current study. First, NAMHS focused on adolescent mental health with the current study exploring the links between ACEs and mental health. The paper provides the baseline findings for the association between ACEs and mental disorders, allowing for future analyses to build upon this and investigate other aspects (e.g., the interplay with other risk factors). It did not explore links with other health issues or developmental outcomes. We also have not assessed the broader conceptualization of ACEs, including exposures beyond the immediate social context [51] and the influence of social, genetic, environmental and structural factors that influence the association of ACEs with mental health. NAMHS is a cross-sectional survey, the directionality of the association between ACEs and mental disorders cannot be explored or confirmed—it is possible that the initial onset of the mental disorder preceded any exposure to ACEs. While this is a limitation, the reported findings are in line with longitudinal studies that have found associations between ACEs and poor mental health [46]. In addition, the ACEs questionnaire utilized in NAMHS is only a relatively brief screener and does not delve into the frequency, severity, or duration of these experiences. This may be vital information for policymakers and stakeholders when looking to develop targeted interventions, particularly as the association with mental disorders may vary as a result. Further, the ACEs questions themselves are limited to specific examples of experiences, for example, specific types of sexual abuse perpetrated by adults, yet evidence from child maltreatment study in Australia found that sexual abuse perpetrated by adolescents, especially by those who are or were in a romantic relationship, has considerably increased [52]. However, the ACEs questions utilised in NAMHS have been widely used in other studies, allowing for further comparisons beyond just NAMHS while providing an important initial evidence base for Kenya, Indonesia, and Vietnam.

## Conclusion

Overall, the findings of the current study demonstrate that ACEs are common among adolescents in Kenya, Indonesia, and Vietnam, albeit with significant differences in prevalence between all three countries. Further, these findings show that despite differences in prevalence, ACEs are associated with increased odds of mental disorders in all three countries. As such, prevention or minimization of the number of ACEs experienced by an individual may be an effective approach for reducing the risk of mental disorders in adolescence. Further, these data provide baseline prevalence estimates by which governments and stakeholders can assess the impact of any population-level efforts to reduce the prevalence of ACEs.

## Additional files

Title of data: Table S1:

Table S1: Unweighted numerators (n) and denominators (N) for prevalence of adverse childhood experiences among adolescents in Kenya, Indonesia, and Vietnam reported in Table 1.

Title of data: Table S2:

S2: Percentage of adolescents endorsing adverse childhood experiences in Kenya, Indonesia, and Vietnam.

Supplementary Material 1

## Data Availability

The NAMHS datasets are co-owned by the University of Queensland and each respective in-country lead organization (K-NAMHS: APHRC and UQ; I-NAMHS: UGM and UQ; V-NAMHS: IOS and UQ). Currently, the datasets or analysis of these datasets are available for collaborative work on request to the relevant data owners following an established protocol. Work is currently underway to convert the NAMHS datasets into public use datasets, allowing for wide use of these datasets while ensuring protection of participant privacy, adherence to country-specific legislation, and appropriate use of data. This includes development of accompanying meta-data, inclusive of a codebook, technical manual, and analysis files. The expected launch date for these public use datasets and accompanying meta-data is 2026, with hosting mechanisms currently under development in line with country legislation and ethical requirements.

## Abbreviations

ACEs: adverse childhood experiences
ADHD: attention-deficit/hyperactivity disorder
aOR: adjusted odds ratio
CIs: confidence intervals
DISC-5: Diagnostic Interview Schedule for Children, Version 5
DSM-5: Diagnostic and Statistical Manual of Mental Disorders, Fifth Edition
GAD-7: Generalized Anxiety Disorder-7
HICs: high-income countries
I-NAMHS: Indonesia – National Adolescent Mental Health Survey
JHSPH: John Hopkins Bloomberg School of Public Health
K-NAMHS: Kenya – National Adolescent Mental Health Survey
LMICs: low- and middle-income countries
NAMHS: National Adolescent Mental Health Surveys
PHQ-9: Patent Health Questionnaire-9
UQ: University of Queensland
V-NAMHS: Viet Nam Adolescent Mental Health Survey
WHO: World Health Organization
