# Supplementary material for: The association between adverse childhood experiences and mental disorders among adolescents in Kenya, Indonesia, and Vietnam: Evidence from the National Adolescent Mental Health Surveys
Source: Child Adolesc Psychiatry Ment Health. 2025 Jul 31;19(Suppl 1):86. doi: 10.1186/s13034-025-00919-z (PMC12312255; doi:10.1186/s13034-025-00919-z)
Supplement: Supplementary file 1 — Supplementary Material 1 [file 13034_2025_919_MOESM1_ESM.docx]

Table S1: Unweighted numerators (n) and denominators (N) for the prevalence of adverse childhood experiences (ACEs) among adolescents in Kenya, Indonesia, and Vietnam.

| **ACEs domain**  *Individual ACE item* | **Kenya** | | **Indonesia** | | **Vietnam** | |
| --- | --- | --- | --- | --- | --- | --- |
|  | **n** | **N** | **n** | **N** | **n** | **N** |
| **Physical abuse**   1. *Ever scared that your parents/other adults were going to hurt you badly* | 588 | 5108 | 732 | 5462 | 383 | 5793 |
| **Emotional abuse**   1. *Ever scared/felt really bad because grown-ups called you names* | 1,218 | 5086 | 922 | 5409 | 746 | 5643 |
| **Neglect**   1. *Ever been a time in your life when you were totally on your own* | 931 | 5091 | 482 | 5498 | 418 | 5818 |
| **Emotional neglect**   1. *Ever felt like you are not loved or cared about* 2. *Ever felt like you have no one*   *that protects you* | 1,079  *813*  *624* | 5140  *5093*  *5114* | 1,055  *900*  *513* | 5584  *5424*  *5485* | 820  *622*  *494* | 5890  *5781*  *5786* |
| **Sexual abuse**   1. *Ever touched by an adult in your private parts except when bathing* 2. *Ever had an adult attempt to or forced you to have sexual intercourse* | 539  *382*  *271* | 5111  *5078*  *5091* | 346  *279*  *130* | 5607  *5478*  *5556* | 115  *92*  *33* | 5906  *5821*  *5836* |
| **Parental substance use**   1. *Ever had parents who drank too much alcohol/used drugs and were abusive* | 334 | 5098 | 88 | 5522 | 96 | 5852 |
| **Poor parental mental health**   1. *Ever saw mother/father so sad that they couldn't take care of you* | 859 | 5109 | 370 | 5460 | 415 | 5734 |
| **Domestic violence**   1. *Ever saw your mother being hit, beaten, or threatened* | 747 | 5110 | 245 | 5533 | 273 | 5810 |
| **Parental incarceration**   1. Ever had either of parents be in prison/jail | 428 | 5094 | 42 | 5584 | 51 | 5907 |
| **Household instability**   1. *Ever had family forced to leave home* 2. *Ever a time when family did not have enough food because of money* | 1,923  *297*  *1826* | 5133  *5112*  *5100* | 614  *88*  *564* | 5616  *5582*  *5266* | 398  *55*  *360* | 5913  *5870*  *5565* |
| **One or more ACEs (out of 13 individual ACEs)** | 3,287 | 5149 | 2,312 | 5653 | 1,975 | 5989 |
| **Four or more ACEs (out of 13 individual ACEs)** | 952 | 5149 | 438 | 5653 | 268 | 5989 |

Table S2: Prevalence of adolescents endorsing adverse childhood experiences in Kenya, Indonesia, and Vietnam.

| **Number of ACEs endorsed** | **Kenya,**  **%, (95% CI)** | **Indonesia,**  **%, (95% CI)** | **Vietnam,**  **%, (95% CI)** |
| --- | --- | --- | --- |
| None | 34.2 (31.5-37.0) | 59.8 (55.9-63.6) | 63.1 (59.2-66.9) |
| 1 | 23.0 (21.3-24.8) | 18.0 (16.3-19.8) | 18.6 (16.8-20.5) |
| 2 | 13.4 (12.2-14.7) | 8.9 (7.9-10.0) | 8.2 (6.9-9.8) |
| 3 | 10.1 (9.0-11.4) | 5.7 (4.6-7.1) | 4.9 (3.9-6.1) |
| 4 or more ACEs | 19.3 (17.5-21.2) | 7.6 (6.3-9.1) | 5.2 (4.2-6.3) |
